# A New and Good Move

**Published:** 1878-06

**Authors:** 


### A NEW AND GOOD MOVE.

Drs. Bogue, of New York, Cook, of Brooklyn, Moffatt, of Boston and Daboll of Buffalo, have united in establishing an office at No. 39 Boulevard Haussmann, Paris, France, where one of the partners at least will always be found.

It is the design, in addition to establishing a practice among the residents, to serve those who may be there temporarily, either those of their own patients or friends from this or other countries, as well as those who may be recommended to them by professional friends.

This arrangement, we have no doubt, will be mutually advantageous to all parties concerned, and we can most cordially suggest to our professional brethren of this country, that they may with implicit confidence recommend their traveling patients to the care of either of these gentlemen.

And may a large measure of success attend the enterprise.

